# Light-Mediated Multilevel Neuromorphic Switching in a Hybrid Organic–Inorganic Memristor

**DOI:** 10.1021/acsomega.4c09401

**Published:** 2024-12-17

**Authors:** Ayoub H. Jaafar, Neil T. Kemp

**Affiliations:** School of Physics and Astronomy, University of Nottingham, Nottingham NG7 2RD, U.K.

## Abstract

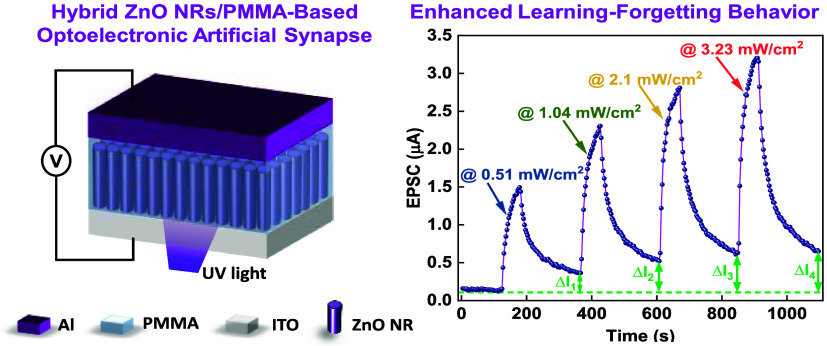

Modulating memristors optically paves the way for new optoelectronic devices with applications in computer vision, neuromorphic computing, and artificial intelligence. Here, we report on memristors based on a hybrid material of vertically aligned zinc oxide nanorods (ZnO NRs) and poly(methyl methacrylate) (PMMA). The memristors require no forming step and exhibit the typical electronic switching properties of a bipolar memristor. The devices can also be switched optically and demonstrate an optically tunable multilevel switching behavior upon illumination with UV light. Additionally, the devices demonstrate high-performance photonic synaptic functionalities, including excitatory postsynaptic current (EPSC), paired-pulse facilitation (PPF), and enhanced potentiation/depression and learning-forgetting characteristics. Notably, after the removal of the UV light, the optoelectronic memristor exhibits a short-term memory due to a persistent photoconductance (PPC) effect. Such a behavior has application in the fabrication of cloned neural networks with pretrained information. The work provides a promising pathway for the fabrication of simple, easy-to-make, and low-cost optoelectronic devices for memory and optically tuned neuromorphic computing applications.

## 1 Introduction

Memristors are promising electronic components for applications in nonvolatile high density and multilevel electronic memories, artificial neural networks, and reconfigurable circuits.^1−4^ Memristors are two-terminal resistive switching devices where the resistance can be switched from a high resistance state (HRS) to a low resistance state (LRS) and *vice versa* upon the application of discrete electrical pulses. As an alternative, the switching between the two resistance states can be achieved using optical pulses, which are practically desirable since light has many advantages, including high bandwidth communication, faster transmission speed, and a noncontact input that does not involve Joule heating.^5−7^ Moreover, tuning the wavelength, polarization, and power intensity of optical stimuli provides dynamic control that can be utilized for enhanced learning in neuromorphic computing applications.^5,8−10^

Recently, optoelectronic memristors have been applied to a range of applications, including highly efficient intelligent computing,^11^ artificial vision systems,^12^ photonic integrated circuits,^13^ and in-sensor reservoir computing systems.^14^ Various structures have been explored such as heterojunction,^6^ planar and vertical architectures,^8^ and various memristive matrices such as two-dimensional (2D) materials,^15^ phase change materials,^13^ oxides,^16^ and organics.^17^ Although these structures and materials have demonstrated the capability for biomimetic memory and learning characteristics, for practical applications, they still face great fabrication and performance challenges. For instance, complex structures using high-cost techniques under vacuum and high temperatures (greater than 810 °C) are commonly used to fabricate the devices, limiting their use for flexible electronic applications.^6,12,13,15^ Also, most reported optoelectronic memristors required a combination of electrical and optical pulses for tuning the learning properties and switching between SET (the transition from HRS to LRS) and RESET (the transition from LRS to HRS) processes.^12^ Moreover, many of the devices required forming steps to initiate memristor switching, involved high SET/RESET voltages, had high power consumption, or utilized complex learning processes that makes difficult the programming of algorithms for artificial neural networks.^8,12^ Hybrid organic–inorganic materials, however, are promising candidates for fabrication of optoelectronic memristors since they combine the electronic characteristics of semiconductors with the solution processing advantages of organic materials, such as low temperature processing, vacuum-free, low-cost fabrication using spin coating process, and large-area coverage at low costs on rigid and flexible substrates.^18−21^ Hybrid materials-based optoelectronic memristors have demonstrated not only excellent properties, such as reduced power consumption, ultralow operation SET and RESET voltages, high on/off ratios, multilevel switching, and mechanical flexibility,^22−24^ but also suitability to optically tunable artificial synapses for the future neuromorphic computing applications.^25−30^

ZnO in the form of zero-dimensional (0D) (nanoparticles),^18,26,31^ one-dimensional (1D) (nanowires/nanorods),^32−34^ and two-dimensional (2D) (thin films)^35−37^ have been used to fabricate memristor devices. The switching mechanisms and resistive switching characteristics of these devices depend on the form of the ZnO material. For example, devices based on ZnO thin films and ZnO nanoparticles have shown filamentary-like switching properties with abrupt switching between HRS and LRS, resulting in a large ON/OFF ratio of over 2 orders of magnitude.^31,38^ Such a digital-type behavior is useful for nonvolatile memory applications,^39^ but the stochastic nature of the filament formation, i.e., random position, size, and orientation, results in variable and nonuniform resistive switching properties in the SET and RESET voltage, resistance of the ON and OFF state, and device-to-device and cycle-to-cycle variability.^39^ These factors are further influenced by postdeposition processes, such as exposure to elevated temperatures during packaging, doping of ZnO to enhance resistive switching characteristics,^40,41^ or adjustments aimed at controlling the forming voltage.^42,43^ On the other hand, devices based on ZnO NRs have shown a homogeneous interface switching with a smooth transition between HRS and LRS.^29^ Such an analogue-type behavior is an essential characteristic to mimic the brain functionalities in neuromorphic computing systems.^29,34^ Compared to other forms of ZnO, the use of ZnO NRs have also the advantage of a high surface area to volume ratio, which facilitates the mobility of defects (oxygen vacancies and zinc interstitials) at surfaces,^44^ confining the switching in one direction and narrowing the switching voltages distribution.^33^ Furthermore, solution-processed ZnO NRs-based devices have also demonstrated forming-free switching due to the formation of large concentrations of defects upon ZnO NRs growth.^29,33^

Here, we report for the first time an optoelectronic memristor based on a hybrid material of ZnO nanorods (NRs) and poly(methyl methacrylate) (PMMA) polymer sandwiched between indium tin oxide (ITO) bottom and aluminum (Al) top electrodes. The device responds to ultraviolet (UV) light with a wavelength of 405 nm. The optoelectronic memristor exhibits a forming-free bipolar resistive switching with a multilevel switching characteristic that is achievable by controlling the light power. Additionally, the optical response of the devices enables dynamic control of synaptic functions, including excitatory postsynaptic current (EPSC), paired-pulse facilitation (PPF), learning-forgetting process, and potentiation and depression characteristics.

## 2 Experimental Section

### 2.1 Synthesis of ZnO NRs

ZnO NRs are conventionally grown by techniques such as pulsed laser deposition,^45^ chemical vapor transport,^46^ and metal–organic chemical vapor deposition.^47,48^ Although capable of forming high quality vertical ZnO NRs, these techniques normally require ultrahigh vacuum equipment and high temperatures, making them slow and expensive to fabricate as well as unsuitable for deposition on lightweight and flexible substrates such as polymers. A desirable alternative is solution-based hydrothermal methods, which are simple, low-cost, and can be used to synthesize vertically aligned ZnO NRs on rigid and flexible substrates at low temperatures and in an ambient atmosphere.^49^

In this work, vertically aligned ZnO NRs were grown using an ultrafast microwave heating method similar to that previously reported.^18,29,50^ The ultrafast microwave method has significantly reduced growth time over conventional hydrothermal hot plate approaches (minutes rather than hours) and has been carefully optimized to eliminate unwanted crystallite formation using a two-step temperature process.^27^ Before the device fabrication, ITO-coated-glass substrates (Delta Technologies) were ultrasonically cleaned in acetone, propan-1-ol, and deionized (DI) water (for 5 min each). Subsequently, the substrates were rinsed with DI water several times and dried with N_2_ gas. Prior to the ZnO NRs growth, a solution consisting of 10 mM zinc acetate dehydrate (98%, Aldrich) in propan-1-ol was first spin-coated at 2000 rpm for 30 s on the substrates to prepare the seed layer for the ZnO growth. The substrates were then annealed on a hot plate in air@350 °C for 30 min. The annealing process is essential to enhance the adhesion of the seed layer to the substrates and to align the crystalline structure of the seed layer for the vertical alignment of NRs. Sequentially, the substrates were immersed in microwave vials containing a solution of 25 mM zinc nitrate hexahydrate (99%, Sigma-Aldrich) and hexamethylenetetramine (HMTA) (99.5%, Sigma-Aldrich) in DI water heated to a growth temperature of 80 °C. After 30 min of growth, the substrates were removed from the microwave, rinsed with DI water, and dried by N_2_.

### 2.2 Device Fabrication

To fabricate the hybrid optoelectronic devices, an ∼100 nm thick PMMA layer was deposited on top of the ZnO NRs using a solution of PMMA (Sigma-Aldrich, 120,000 *M*_w_, dissolved in toluene, 5% by weight) by spin coating at 5000 rpm for 30 s, followed by annealing on a hot plate at 140 °C for 30 min to remove any residual solvent. Finally, 100 nm thick top Al electrodes were deposited by thermal evaporation under vacuum conditions through a shadow mask containing 400 μm diameter circles, giving a device structure of ITO/ZnO NRs/PMMA/Al. Note that a reference device consisting of only ZnO NRs sandwiched between the bottom ITO and top Au electrodes was also fabricated.

### 2.3 Characterization

Scanning electron microscopy (SEM) was used for imaging the ZnO NR thin films. Current–voltage (*I*–*V*) characteristics and optical illumination were carried out using a Keithley 4200A-SCS Parameter Analyzer and a UV laser (Oxxius-405 nm), respectively. For electrical characteristics, the voltage was applied on top Al electrodes while the bottom ITO electrodes were grounded. All of the electrical characteristics were achieved at room temperature in an ambient atmosphere.

## 3 Results

**Figure 1:**
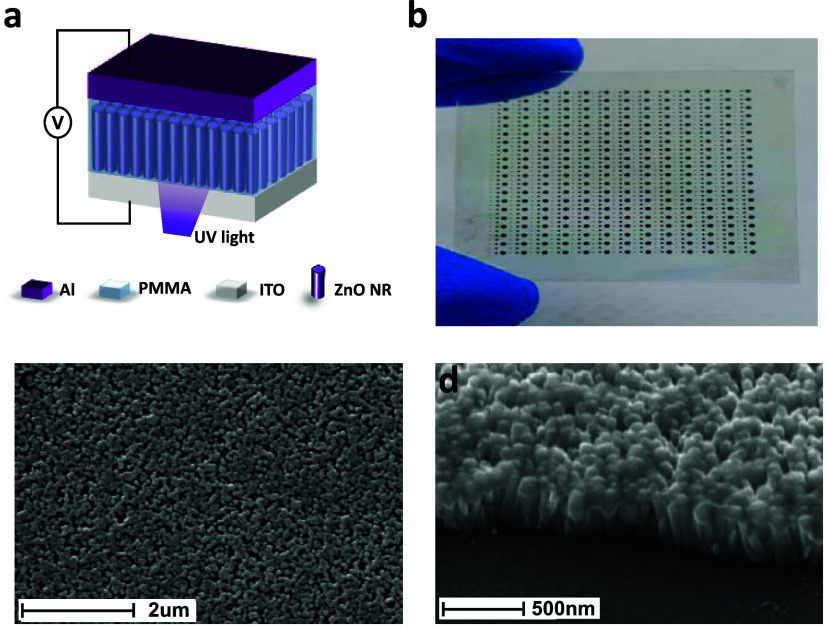
(a) Schematic of the hybrid optoelectronic ZnO NRs/PMMA memristor. (b) Photograph of a completed sample of many optoelectronic memristor devices. (c) Top view SEM image of the ZnO NRs. (d) SEM image of the ZnO NR arrays (sample tilted at 70°).

Figure 1a schematically shows the hybrid organic–inorganic optoelectronic memristor with a structure of ITO/ZnO NRs/PMMA/Al. A photograph of the sample after fabrication showing many optoelectronic memristors is shown in Figure 1b. Top view SEM image showing the surface morphology of the ZnO NRs thin film is presented in Figure 1c. It can be clearly seen that the ZnO NRs with hexagonal structures are closely packed across the substrate. An SEM image with a 70° tilt angle of the ZnO NR arrays grown on an ITO-coated glass substrate is shown in Figure 1d. The length and diameters of ZnO NRs are about 200 and 40–60 nm, respectively. Note that the length of ZnO NRs appears longer due to the 70° sample tilt used in the SEM. The figure shows that very well packed and vertically aligned ZnO NRs were grown on the substrate, demonstrating the high-quality ZnO achieved via an ultrafast microwave growth technique.

**Figure 2:**
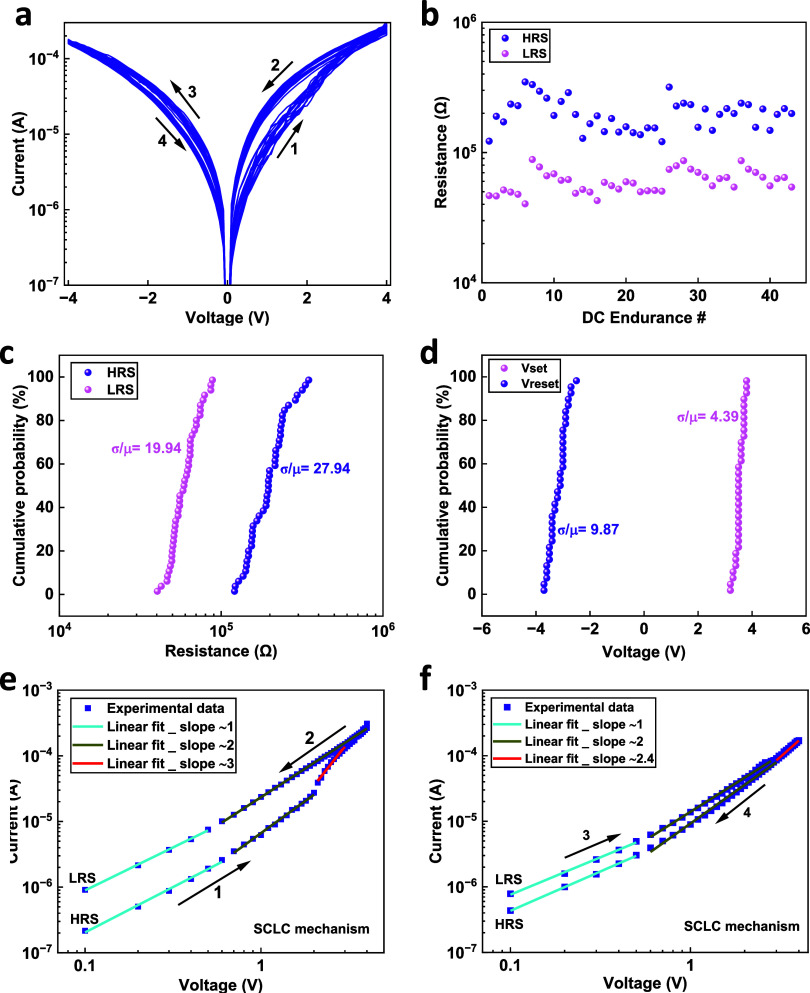
(a) *I*–*V* characteristics of ITO/ZnO NRs/PMMA/Al optoelectronic memristor. (b) Endurance of an optoelectronic device at a read voltage of 0.5 V showing reproducible HRS and LRS behaviors. (c) Cumulative probability of HRS and LRS and (d) *V*_SET_ and *V*_RESET_. (e) and (f) Experimental data and fitted *I*–*V* curves on a log–log scale showing a fit for the space-charge-limited current (SCLC) mechanism for the SET and RESET processes, respectively.

Current–voltage (*I*–*V*) measurements were used to investigate the electronic switching characteristics of the hybrid optoelectronic devices. Figure 2a shows successive *I*–*V* sweeps for a device swept under an external applied voltage of ±4 V. Note that no forming process was needed to initiate resistive switching in the device as previously reported in ZnO NR hybrid memristor devices, indicating the presence of conducting defect pathways within the ZnO NRs.^18,29,50^ This is expected since a large concentration of defects are formed during the formation of the ZnO material by the rapid hydrothermal growth method.^51^ The direction of the current sweep is indicated by arrows. The plot shows that the device has a typical bipolar pinched hysteresis loop with a positive SET and negative RESET. The device is initially in an HRS. Upon application of a positive sweep voltage (from 0 to +4 V), the device is switched from the HRS to the LRS at a SET voltage of approximately 3 V. The device stayed in its LRS during the backward sweep voltage (from +4 to 0 V). However, reversing the applied voltage polarity switched the device from the LRS to HRS at a RESET voltage of approximately −3 V. Successive *I*–*V* sweeps for another optoelectronic memristor device showing a reliable and reproducible resistive switching behavior are shown in Figure S1a.

To examine the operational reliability of our hybrid ZnO NRs/PMMA optoelectronic memristor, the resistive switching performance characteristics, including DC endurance and cumulative probability for the ON/OFF resistance ratio, were analyzed. As can be seen in Figure 2b, the device exhibits a stable endurance performance in the DC mode for both the HRS and LRS, in which no degradation was observed for more than 40 cycles. Figure 2c shows the high uniformity of cumulative probability distribution for both states with the coefficients of the variation (σ/μ, where σ is the standard deviation and μ is the mean value) calculated to be 27.94 and 19.94% for HRS and LRS, respectively. Furthermore, the operational switching voltages uniformity was also examined. High uniformity cumulative distributions were observed for *V*_SET_ and *V*_RESET_, as shown in Figure 2d, where the coefficients of variation were 4.39 and 9.87%, respectively. Endurance performance tests for another optoelectronic memristor device are shown in Figure S1b.

The conduction mechanism of the hybrid optoelectronic device was investigated by plotting the positive and negative parts of the *I*–*V* sweep on a log *I*–log *V* scale, as shown in Figure 2e,f, respectively. The fit agrees with a conduction mechanism governed by the space-charge-limited current (SCLC) mechanism. Initially, for the positive part of the *I*–*V*, SET process (Figure 2e), from 0 to 0.6 V (with the device in the HRS), a linear relationship with a slope of about 1 is present, indicating an Ohmic conduction process, which arises from thermally generated charge carriers.^52^ At higher applied bias (0.65 ≤ *V* ≤ 2), the slope changes to about 2, and the current shows the voltage square dependence (*I* ∝ *V*^2^), which can be attributed to the trap-controlled space-charge-limited current (TC-SCLC), as described by Mott–Gurney law and observed in other ZnO systems:^26,53^, where *J* is the current density, ε is the dielectric constant, μ is the free carrier density, *V* is the applied voltage, and *d* is the film thickness. In the region of 2.1 ≤ 3.2 V, a much steeper rate of current increase occurs with a slope of 3. This indicates that many of the traps are filled, and the conduction in this region is similar to trap-filled space-charge-limited current (TF-SCLC), resulting in switching the device from the HRS to the LRS. In the case of the LRS, the conduction mechanism is dominated by two regions: Ohmic conduction at low applied voltage, featuring the linear dependence of current with applied voltage (*I* ∝ *V*), and SCLC at higher voltage, where the current shows the voltage square dependence, (*I* ∝ *V*^2^). For the negative part of the *I*–*V*, RESET process (Figure 2f), the conduction mechanism in the high applied voltage region switches from TF-SCLC in the LRS to TC-SCLC in the HRS with Ohmic conduction being dominated at low applied voltages for both states.

To better evaluate the impact of the additional PMMA layer on the optoelectronic memristor switching properties, including the ON and OFF currents, the power consumption, and the ON/OFF resistance ratio, a comparison study between two device structures was made. This involved a ZnO NRs only memristor device and a hybrid ZnO NRs/PMMA memristor. Figure S1a shows the *I*–*V* curves for these two types of devices. The plot shows both the ON and OFF currents decreased by 3 orders of magnitude upon adding the PPMA layer, resulting in much lower power consumption. Moreover, the ON/OFF resistance ratio increases from 1.3 for the ZnO NRs device to 4.1 for the hybrid device. The results indicate that PMMA acts as a barrier to isolate the top and bottom electrodes, preventing any potential short-circuit effect while also enhancing the yield and stability of devices.^44^ All of these comparisons clearly suggest that the switching performance of the inorganic ZnO NRs optoelectronic memristor can be significantly improved by incorporating the PMMA organic layer.

**Figure 3:**
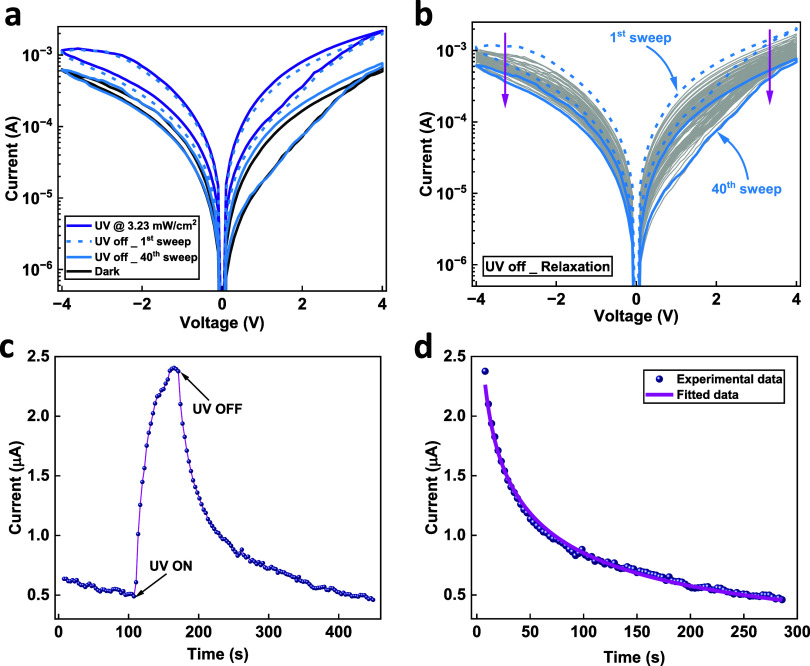
(a) *I*–*V* curves for the hybrid ZnO NRs/PMMA optoelectronic memristor under dark (black curve) and UV illumination at 3.23 mW/cm^2^ (purple curve), followed by a series of 40 sweeps after the UV light had been removed (light blue curve). (b) Same *I*–*V* curves but just showing the relaxation of the device (40 sweeps) to the initial state after switching off the light. (c) Typical photoresponse characteristics of a hybrid device illuminated at 1.04 mW/cm^2^ for 1 min. (d) Fitting for the relaxation process and time for the device to return to its initial state after switching the light off.

The optoelectronic response of the hybrid ZnO NRs/PMMA memristor is explored in Figure 3a. In dark conditions, the device electrically switches between the HRS and the LRS (see black curve in Figure 3a). Upon illumination with UV light (UV at 3.23 mW/cm^2^), a photoconductance (PPC) effect occurs, which causes the *I*–*V* curve to shift to a higher current (about 1 order of magnitude) for both the SET and RESET states of the device (purple curve). In contrast, the ZnO NRs-only device (no PMMA) showed a much lower photocurrent response than the hybrid device upon UV illumination; see Figure S2. Interestingly, these states do not easily depopulate once the UV light is removed but can be encouraged to do so through the application of multiple *I*–*V* sweeps, as shown by the 40 successive sweeps in Figure 3a,b. Note that the time for the 40 sweeps was about 700 s. The effect was reproducible in other similar devices, as can be seen in Figure S3a,b.

To further investigate the relaxation time, a transient photoresponse measurement was performed. Figure 3c demonstrates the typical photoresponse characteristics of the hybrid optoelectronic device under UV illumination at 1.04 mW/cm^2^ for a pulse width of 1 min. The current (read@0.2 V) exhibits a sharp increase under UV illumination, showing a photocurrent effect. However, on the removal of UV light, the current gradually decays to its initial level, with a decay time of about 270 s, displaying a persistent photoconductance (PPC) effect. We debate the possible cause for the photoresponse and PPC in the Discussion section.

The decay curve (in Figure 3c) after the removal of the UV light was fitted using a stretched-exponential based function, *I*(*t*) = *I*_0_ *e*^[−(*t*/τ)^β^]^,^54^ as shown in Figure 3d. Here, *I*(*t*) is the current at a given time, *I*_0_ is the current level at *t* = 0, τ = 5 s is the characteristic relaxation time, and β = 0.25 is the stretch index. The fitted relaxation time was found to be 5 s. This relaxation effect is desirable for temporal processing time or short-term memory (STM) for optically tunable neuromorphic and reservoir computing applications.^55^

**Figure 4:**
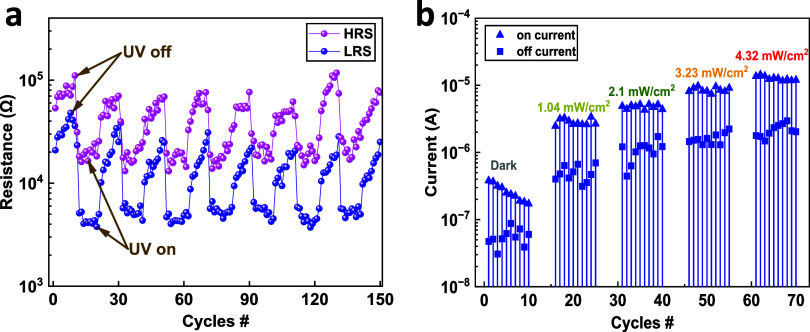
(a) Optical modulation of the device resistances for a hybrid ZnO NRs/PMMA memristor by illumination with UV light at 4.32 mW/cm^2^. (b) Optical modulation of the electrically switchable on and off states for a hybrid ZnO NRs/PMMA memristor upon illumination with different UV light powers, achieving multilevel switching states. The data were extracted from *I*–*V* sweeps at 1 V.

For optoelectronic memristors, investigating light-mediated memory capabilities is important. Figure 4a illustrates an optical cycling between illumination at 4.32 mW/cm^2^ and dark conditions for the LRS and HRS of a device with electrical pulses of ±3 V (at a pulse width of 100 ms) and read voltage pulse of 0.1 V. The plot indicates that optical cycling can be used to achieve a multilevel switching effect. In addition, the multilevel switching effect can also be achieved for the HRS and LRS upon varying the light power, as shown in Figure 4b. Such a behavior suggests that the device has a potential application in high-density multistate storage memory.^2^

**Figure 5:**
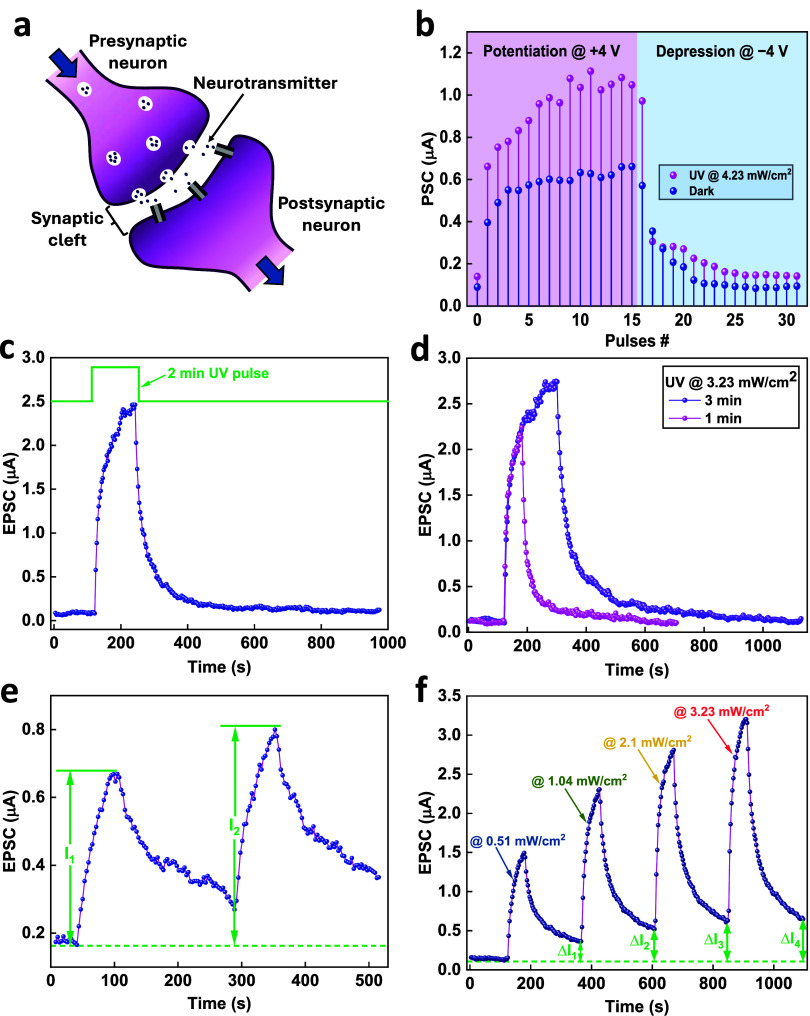
(a) Schematic representation of synaptic plasticity of a hybrid ZnO NRs/PMMA optoelectronic memristor. (b) Optical modulation of the potentiation and depression in a hybrid ZnO NRs/PMMA memristor. (c) EPSC behavior of the optoelectronic synapse, read@0.2 V. (d) EPSC behavior of the optoelectronic synapse as a function of UV pulse width, read@0.2 V. (e) PPF behavior of the optoelectronic synapse under paired UV pulses, read@0.2 V. (f) Enhanced learning-forgetting behavior@different UV powers, read@0.15 V.

Further, the potential of the optoelectronic device in controlling the neuromorphic learning properties by optical means was investigated. As a two-terminal device, memristors can be used as biological synapses whose synaptic weights can be dynamically modified and stored upon application of external electrical or optical stimuli. Figure 5a shows a schematic illustration of a biological synapse. The effect of UV illumination (@4.32 mW/cm^2^) on the synaptic plasticity of the device is investigated. Figure 5b shows how light (either on or off) can impart different properties to the artificial synapse. The potentiation and depression effect was achieved through the application of 15 successive positive pulses (@+4 V), followed by 15 successive negative pulses (@–4 V) with a pulse width of 100 ns and pulse interval of 1 ns, and a read voltage of 0.15 V. This was done when the device was either illuminated (pink data) or not illuminated (blue data). It can be seen that light illumination enhances the potentiation and depression effect.

A more advanced photonic synaptic function is the excitatory postsynaptic current (EPSC) behavior, which occurs upon transferring light signals as presynaptic stimuli to electrical signals as postsynaptic current in an optoelectronic synapse.^56^ This behavior is emulated by our optoelectronic synaptic devices. As shown in Figure 5c, a typical EPSC behavior can be achieved under a UV pulse at 3.23 mW/cm^2^ with a pulse width of 2 min. The EPSC quickly increases upon illumination and then gradually decreases after removing the UV light due to the photocurrent and the PPC effect, respectively. The intrinsic volatile characteristic of the photocurrent demonstrates the short-term memory (STM) behavior, confirming that our hybrid optoelectronic synapse has both sensing and memory functionalities.^57^ Additionally, modifying the pulse width can significantly modulate the EPSC behavior, as presented in Figure 5d. The plot shows that the longer pulse width leads to stronger synaptic weights and longer decay time. Further EPSC behaviors under a UV pulse@1.04 mW/cm^2^ for 1 min and@3.23 mW/cm^2^ for 3 min for two optoelectronic synaptic devices are shown in Figure S3c,d, respectively.

One of the most representative behaviors of STM is paired-pulse facilitation (PPF), which is a phenomenon observed in neuroscience, where the response of a neuron to a second synaptic stimulus is enhanced following an initial stimulus. PPF is thought to play a role in short-term information processing, as it allows synapses to temporarily boost their signaling strength after a series of rapid excitatory pulses. It also allows for enhanced synaptic transmission during periods of high-frequency activity. By utilizing optical control, such a system could allow a higher-level task manager to selectively activate specific subsections of a larger network to initiate learning or perform high-priority tasks on demand. To establish this effect, we applied two successive light pulses (1.04 mW/cm^2^, 1 min pulse width, 3 min pulse interval) to the synapse, as illustrated in Figure 5e. The response current *I*_2_ under the second pulse was higher than the response current *I*_1_ under the first pulse. The PPF can be calculated by the following equation: , and it was found to be 20%.

The learning-forgetting process is one of the basic functions of the human brain. Information that is initially stored in temporary memory decays unless a reinforcement process occurs through a repetitive learning process.^61^ This process is emulated by our optoelectronic synapse and is presented in Figure 5f. Here, the enhanced learning and forgetting processes correspond to the UV pulses (1 min pulse width) and darkness (3 min pulse interval), respectively. After repeating the learning-forgetting processes at four different UV powers, the EPSC of the synapse increases (Δ*I*_4_ > Δ*I*_3_ > Δ*I*_2_ > Δ*I*_1_), indicating that relearning the previously stored information can strengthen the memory ability. Further enhanced learning and forgetting processes corresponding to the UV pulses at different powers for another synaptic device can be seen in Figure S4. Such enhanced learning and forgetting processes could have applications in emulating the brain’s ability to perform consolidation, which is a process used to strengthen and stabilize new information. This is thought to occur during sleep and involves moving memories from short-term to long-term memory storage. Optical control of the learning-forgetting process could reinforce regions storing short-term memories, extending their retention and allowing more time for the eventual transfer to long-term memory. Potentially, this approach could also enable the direct conversion of entire regions to long-term storage, eliminating the need to transfer information between separate memory systems.

## 4 Discussion

In this section, the mechanism for the optical photoconductance and PPC effect is first discussed. It is known that the photoconductance effect in ZnO is dominated by the role of adsorbed oxygen on the surface of the ZnO material, as demonstrated by measurements of the photoconductance of ZnO in vacuum, air, and nitrogen environments.^62^ Upon illumination by UV light, photogenerated holes from the exciton liberate the chemisorbed oxygen molecules, releasing them from the surface of the ZnO while the photoexcited electrons in the conduction band increase the conductance of the material. The rate of increase has been found to be entirely dependent on the rate of physisorption process.^57,63^ Upon removal of the UV light, oxygen molecules readsorbed on the ZnO surface passivate the surface defect states with a rate fully dependent on the readsorption rate of the oxygen molecules. Often, a PPC effect is also observed, which is attributed to a metastable state between a shallow and a deep energy state. Such a defect state is the deep unknown (DX) center, which occurs when a shallow donor converts into a deep donor because of a large lattice relaxation.^62^ In this case, the creation of a thermally activated barrier prevents the recapture of electrons by the DX center, leading to long PPC. Typical relaxation times range from several hours to several days. The persistence effect is also dependent on the environmental conditions (i.e., vacuum, nitrogen, or air), since it is dependent upon having a reservoir of oxygen molecules and the porous nature of the ZnO material.^64,65^ The persistence effect can be modulated through encapsulation of the ZnO in a polymer,^66^ which inhibits the release of the oxygen into the environment, allowing it to be more easily readsorbed back into the defect states.

Based on these known effects in ZnO, we propose that a similar process occurs in the ITO/ZnO NRs/PMMA/Al optoelectronic memristors. UV illumination causes the release of oxygen molecules from the surface of the ZnO NRs into the PMMA material. However, it is expected that most of the oxygen stays within the vicinity of the ZnO NRs, causing the oxygen-rich PMMA to act as a reservoir for the readsorption of oxygen when the UV light is removed. The conductance of the device is improved because of the release of electrons into the conduction band, as observed by an increased current for both positive and negative potentials in the *I*–*V* sweeps and also by the transient photoresponse measurement. When the UV light is removed, the PPC effect occurs upon the oxygen molecule readsorption process at the ZnO NR surface.

The PPC mechanism is however still greatly debated, with some attributing it to the readsorption of oxygen molecules on the ZnO surface, while others suggest it is linked to the narrowing or widening of the interfacial region caused by ionization or neutralization of intrinsic defects, such as oxygen vacancies, within the ZnO material.^67,68^

Compared with earlier studies on optical switching effects in ZnO, our hybrid inorganic–organic device architecture and ZnO fabrication method offer several distinct advantages. From a fabrication standpoint, the use of wet chemical techniques, rather than magnetron sputtering or molecular beam epitaxy, provides significant commercial benefits. Wet chemical methods enable low-cost, high-throughput device production under ambient conditions, avoiding the need for high-temperature or vacuum environments. By encapsulation of ZnO in PMMA, oxygen loss to the environment is prevented during the PPC mechanism, ensuring device operation is not dependent on the environmental conditions or effects of chip packaging. Furthermore, PMMA encapsulation offers a potential avenue for tuning the retention time of the volatile PPC memory state. By adjusting the molecular weight of the polymer, which modifies the oxygen diffusion rate, the retention time should be able to be controlled.

Compared with recent reports, our device demonstrates noticeable differences in operation and performance specifications. Recent studies of bare ZnO, without PMMA encapsulation, in the form of 1D nanowires and 2D thin films have been reported. In the case of Zn nanowire-based devices (Ag/ZnO/Ag planar structure),^32^ no resistive switching effect was observed under dark conditions, but resistive switching appeared upon UV illumination (365 nm) only when the voltage exceeded 2 V, indicating the need for a forming step. Following UV light removal, the memory state was slow to recover and did not fully return to its initial state after more than 10 min.

In another study, consisting of a magnetron sputtered ZnO thin film with a vertical Pt/ZnO/Pt structure,^69^ the device showed a full recovery after UV illumination but had a much smaller photocurrent response. Specifically, there was an ∼30% increase in the photocurrent after UV exposure of 100 s, compared to the ∼400% increase observed in our device for the same exposure time (Figure 3c). Furthermore, the recovery time of the sputtered thin-film ZnO device was dependent on a double exponential function, indicating two distinct mechanisms, whereas our device demonstrated a much faster recovery, described by a single exponential function, indicating a single dominant mechanism.

It was found that the PPC effect in our devices varies from device to device, which could be attributed to the variation in the PMMA film thickness across the sample. Note that we exclude the PMMA response to UV light as it is transparent to UV light. However, the PMMA plays a key role in preventing interaction with external gases and passivating the electron states of ZnO associated with dangling bonds at the surface.^70^ The PMMA also enhances the response speed in comparison with bare (uncoated) ZnO NRs and nanowires.^32,57,69,71,72^ Such a behavior has an important application in setting the initial value of weights in neural networks to predefined values^8^ and UV detection applications.^57^

Although the response and decay times are relatively slow, neural processing occurs over very broad time scales in biological systems, typically ranging from milliseconds (e.g., action potentials) to minutes (short-term plasticity) and even hours, days, and years (long-term memory). It is expected that such a broad range of time scales is important to carry out a variety of complex neural functions.^58−60^ These can be short-term transient computational processes, such as noise filtering or temporal pattern recognition, or long-term processes optimized for encoding stable patterns and knowledge over time. The latter is particularly important for reinforcement learning, adaptive behavior, and prediction. Neuromorphic systems with multiscale time constants also enable adaptability, allowing systems to simultaneously carry out multiple tasks of both a short-term or long-term nature or allowing a system to modify its network so that it can dynamically switch between different computational tasks. Such dynamical systems can evolve toward greater intelligence and energy efficiency. Relaxation processes, which negate the need for a reset (erase) step, also play an important role in the evolutionary dynamics of these systems.

In summary, based on the experimental results presented in this work, our optoelectronic synaptic devices offer proof of concept for neuromorphic computing applications. From a fabrication perspective, the solution-based processing methods used, specifically the ultrafast microwave-assisted hydrothermal technique, enable the production of high-density optoelectronic devices on both rigid and flexible substrates at a much lower cost compared with conventional semiconductor fabrication methods. However, traditional semiconductor techniques typically offer greater precision in purity and fabrication, allowing for a higher scalability. At this stage, it is uncertain whether the ZnO/PMMA devices can be scaled down to a single ZnO nanorod while maintaining their optoelectronic switching performance. Another challenge to overcome is the variability in the response speed of the PPC effect, which differs between the devices. It remains unclear whether these variations are caused by differences in material properties or structural morphology such as PMMA thickness or ZnO length/diameter variations.

## 5 Conclusions

This work demonstrates an optoelectronic memristor based on a hybrid material consisting of ZnO NRs and PMMA. The hybrid optoelectronic memristor shows desirable characteristics, including forming-free operation and compliance-free bipolar switching. Besides electronic memristor switching in dark conditions, the device responds to UV light, which enables tunability of the memristor state and a range of accessible multilevel states depending on the light intensity. Optical stimulation was importantly found to cause a prolonged short-term memory effect, called PPC, which can be utilized to implement more complex neuromorphic computing capabilities. A range of essential synaptic behaviors, including EPSC, PPF, potentiation/depression, and learning-forgetting processes, were successfully emulated in the hybrid optoelectronic memristor. These results pave the way for the fabrication of optically tunable memristor devices by using a cost-effective wet chemical solution processing strategy. The devices’ multilevel memory storage capabilities and demonstration of key synaptic behaviors hold significant potential for applications in neuromorphic computing and in-sensor computer vision.

## Supporting Information Available

*I*–*V* curves for a hybrid ZnO NRs/PMMA-based optoelectronic memristor and for ZnO NRs-based device showing a comparison between the two device types; *I*–*V* curves for a ZnO NRs-based device under dark and UV conditions; *I*–*V* curves and typical photoresponse characteristics of hybrid devices illuminated at different UV powers showing the relaxation processes after switching off the light; and enhanced learning-forgetting behavior at different UV powers (PDF)
