# Supplementary material for: Light-Mediated Multilevel Neuromorphic Switching in a Hybrid Organic–Inorganic Memristor
Source: ACS Omega. 2024 Dec 17;9(52):51641–51. doi: 10.1021/acsomega.4c09401 (PMC11696397; doi:10.1021/acsomega.4c09401)
Supplement: Supplementary file 1 — ao4c09401_si_001.pdf [file ao4c09401_si_001.pdf]

Supporting Information

Light-Mediated Multi-Level Neuromorphic Switching in a Hybrid Organic-Inorganic Memristor

Ayoub H. Jaafar,[a] and Neil T. Kemp[a,*]

[a] School of Physics and Astronomy, University of Nottingham, Nottingham, NG7 2RD, U.K.

*Author to whom correspondence should be addressed. Electronic mail:

neil.kemp@nottingham.ac.uk

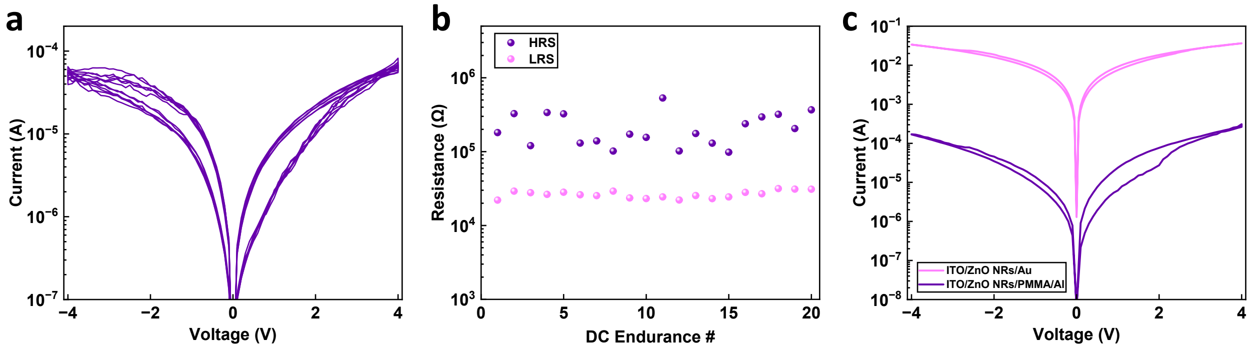

Figure S1. a) *I-V* curves for a hybrid ZnO NRs/PMMA optoelectronic memristor, showing repeatable and uniform resistive switching performance. b) Endurance of an optoelectronic device at a read voltage of 0.5 V showing reproducible HRS and LRS behavior. c) *I-V* characteristics

showing a comparison of two device structures; ITO/ZnO NRs/Au, and ITO/ZnO NRs/PMMA/Al. The hybrid device (containing insulating PMMA) has greatly increased resistance.

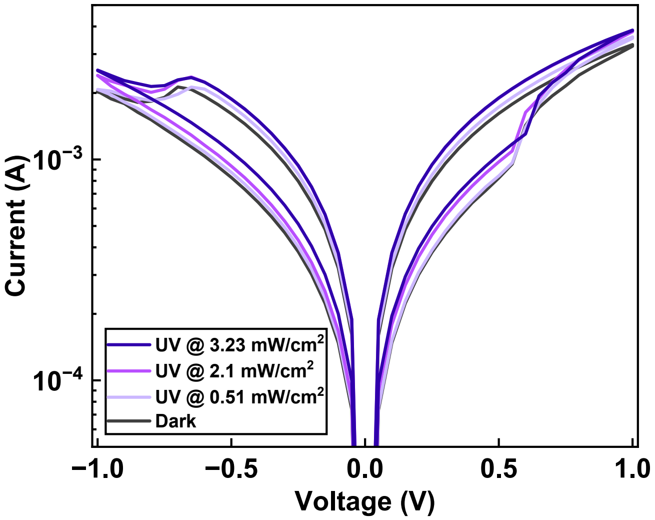

Figure S2. *I-V* curves for an ITO/ZnO NRs/Au optoelectronic memristor device under dark and UV illumination at different powers conditions. The plot shows that the device responds to the UV light. However, the response is small in magnitude in comparison to the hybrid device, indicating the potential use of PMMA layer to enhance the optoelectronic effect. The small UV response of the device can be attributed to its initial high conductance (in dark conditions).

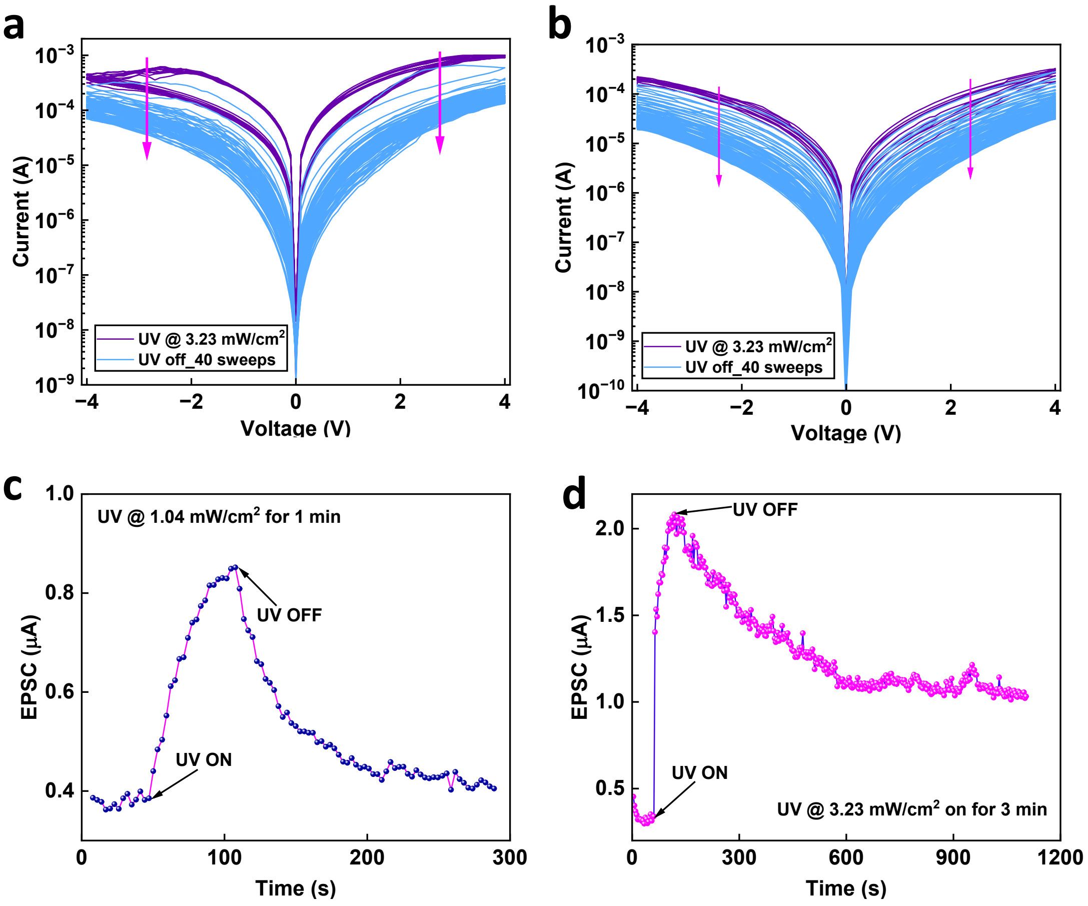

Figure S3. a) and b) *I*-*V* curves for different hybrid ZnO NRs/PMMA optoelectronic memristors under UV illumination at 3.23 mW/cm^2 (purple curve) followed by a series of 40 sweeps after the UV light had been removed (light blue curve). c) The typical photo-response characteristics of a hybrid device illuminated @ 1.04 mW/cm^2 for 1 min. d) The typical photo-response characteristics of a hybrid device illuminated @ 3.23 mW/cm^2 for 3 min. The current in Figure c and d was read @ 0.2 V. The figure shows that the PPC effect can be varied from device to device, which can be attributed to the variation in the PMMA film thickness across the sample as well as the amplitude and time of the applied UV power.

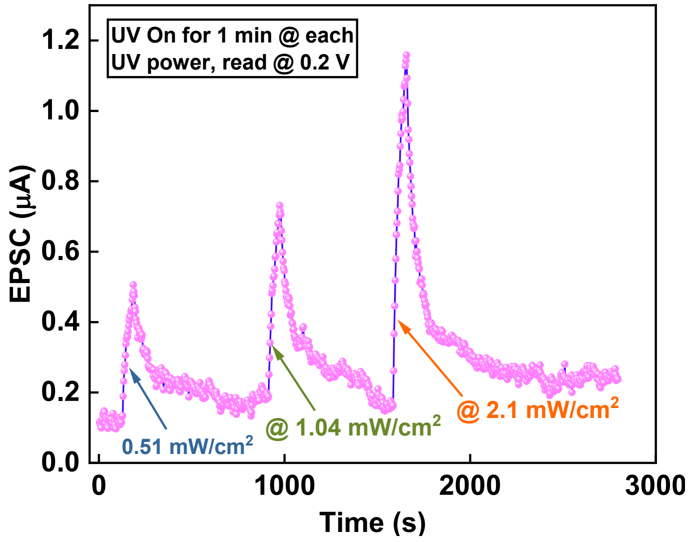

Figure S4. The enhanced learning-forgetting behavior @ different UV powers, read @ 0.2 V. The enhanced learning and forgetting processes correspond to the UV pulses (1 min pulse width) and darkness (12 min pulse interval), respectively. Although, the forgetting process took a long time (12 min or more), the next enhanced learning process can be achieved quickly once the next UV pulse at different power is applied, demonstrating the change from STP to LTP.
